# Mother-to-child transmission and gestational syphilis: Spatial-temporal epidemiology and demographics in a Brazilian region

**DOI:** 10.1371/journal.pntd.0007122

**Published:** 2019-02-21

**Authors:** Joyce Marinho de Souza, Rogério Giuffrida, Ana Paula Marques Ramos, Glilciane Morceli, Camila H. Coelho, Marcus Vinícius Pimenta Rodrigues

**Affiliations:** 1 Faculdade de Ciências da Saúde, Biomedicina, Universidade do Oeste Paulista, Presidente Prudente, SP, Brazil; 2 Laboratório de Biologia Molecular de Microrganismos, Universidade Estadual Paulista, Londrina, PR, Brazil; 3 Programa de pós-graduação em Meio Ambiente e Desenvolvimento Regional, Universidade do Oeste Paulista, Presidente Prudente, SP, Brazil; 4 Programa de pós-graduação em Ciência Animal, Universidade do Oeste Paulista, Presidente Prudente, SP, Brazil; 5 Mestrado em Ciências da Saúde, Universidade do Oeste Paulista, Presidente Prudente, SP, Brazil; 6 Laboratory of Malaria Immunology and Vaccinology, DIR, NIAID, NIH, Bethesda, MD, United States of America; University of Connecticut Health Center, UNITED STATES

## Abstract

Syphilis is a Sexually Transmitted Infection (IST) with significant importance to public health, due to its impact during pregnancy (Gestational Syphilis—GS); especially because syphilis can affect fetus and neonates’ development (mother-to-child transmission—MTCT of syphilis), by increasing susceptibility to abortion, premature birth, skeletal malformations, meningitis and pneumonia. Measures to control and eliminate MTCT of syphilis have failed on the last few years in Brazil and this research aimed to identify the seasonality of notified cases of syphilis in a region of São Paulo state. The studied region, Pontal do Paranapanema, comprises 32 cities located in the West of São Paulo state, in Brazil. Data collected from the National System of Aggravations and Notification (SINAN) website was used to calculate the incidence rate of GS and MTCT. The incidence rate of GS was acquired dividing number of cases by number of women in each municipality and MTCT using number of live births in each year (from 2007 to 2013) in each municipality. This result was then, standardized multiplying incidence rate by 10,000 and expressed as incidence/10,000 women or live births, for GS and MTCT, respectively. To identify possible endemic/epidemic periods, a control diagram was performed using the standard deviation (SD) of incidence rate. Thematic maps representing the spatial distribution of incidence rates were constructed using a Geographic Information System software (GIS, based on cartographic vector available on the Brazilian Institute of Geography and Statistics (IBGE) website. Eighty cases of GS and 61 cases of MTCT were notified in the studied region. An increase of GS notification was detected in the Pontal do Paranapanema in 2011 followed by an increase in number of MTCT cases in the subsequent year, suggesting inefficacy in the treatment during gestational period. Most of those cases were reported on February and November which suggested seasonality for this IST in the region. The control diagram, based on the inputs collected from SINAN, showed no endemic period; however, the most susceptible month to happen an endemic event of GS and MTCT was February. Our study provided a new methodology to understand the syphilis dynamics as a potential tool to improve the success of future measures to control and possibly eliminate MTCT of syphilis.

## Author summary

Syphilis is a sexually transmitted infection that can be transmitted to child during pregnancy, or postpartum, by an infected mother. Syphilis in children can interfere in the development and, in some cases, may lead to death. We analyzed cases of syphilis during pregnancy and mother-to-child transmission of syphilis from 2007 to 2013 occurred in a Brazilian region. We used a public online website provided by the Brazilian government to obtain information about cases, generate map locations and design a transmission pattern in the region. During the period investigated, we identified 80 cases of syphilis in pregnant woman and 61 related to mother-to-child transmission, both notified mainly during Summer (from November to February). Children infection might be related to incorrect treatment of mother and the partner. Not only the mother must be treated but the partner as well, otherwise women may be infected during pregnancy, transmitting syphilis to fetus. This study may help in the establishment of measures to control and eliminate mother-to-child transmission of syphilis.

## Introduction

*Treponema pallidum* is a spirochete bacterium that causes syphilis, a sexually transmitted infection (STI) transmitted due to contact with infected lesions. However, the transmission is not limited to this route since pregnant women infected with the microorganism can develop gestational syphilis (GS) what in its turn may lead to hematogenic transmission of *T*. *pallidum* to fetus, causing mother-to-child transmission (MTCT) of syphilis [1,2]. In Brazil 37,436 pregnant women and 20,474 children were notified with syphilis in 2016 [3]. These numbers represent an increase of 10% in comparison to 2015, and 40% in comparison to 2010 [3].

In despite of implementation of health care programs for pregnant women aimed to control and eliminate GS and MTCT in the country, syphilis incidence has increased significantly and lack of diagnosis may have led to underreported GS and MTCT cases [4,5].

Aiming to overcome those health systems failures, innovative approaches to study GS and MTCT are necessary. Identification of seasonality and incidence as well as specific factors related to pregnant women and child infection, may help control and prevent GS and MTCT outbreaks. According to Bertolozzi and colleagues (2009), the vulnerability of a region to a particular disease is related to socioeconomic and environmental factors that involve individuals [6]. Pontal do Paranapanema is one of the most vulnerable regions of the state of São Paulo, with low family income, high school dropout rates and inadequate sanitation and health conditions [7,8], thus characterizing a region of vulnerability to infectious diseases.

Epidemic and seasonal patterns of infectious diseases are widely used by public health systems to monitor communicable diseases, especially vector-borne infections [9,10]. Although the study of seasonal dynamics of STIs is an approach extensively used to characterize other diseases [11], it is not frequently used to understand GS and MTCT. Aiming to overcome the need for epidemiological studies to understand GS and MTCT in the Pontal do Paranapanema, in this study we described a complex analysis of this region regarding to gestational and MTCT of syphilis. We included spatial distribution of GS and MTCT seasonal and epidemic patterns, as well as characteristics of pregnant and child diagnosed and notified with this infection from 2007 to 2013, living in the Pontal do Paranapanema.

Our data describes the behavior of the disease transmission and suggest improvement in prevention and screening campaigns to effectively prevent syphilis in Brazil.

## Methods

### Data collection and statistics analyses

**Fig 1:**
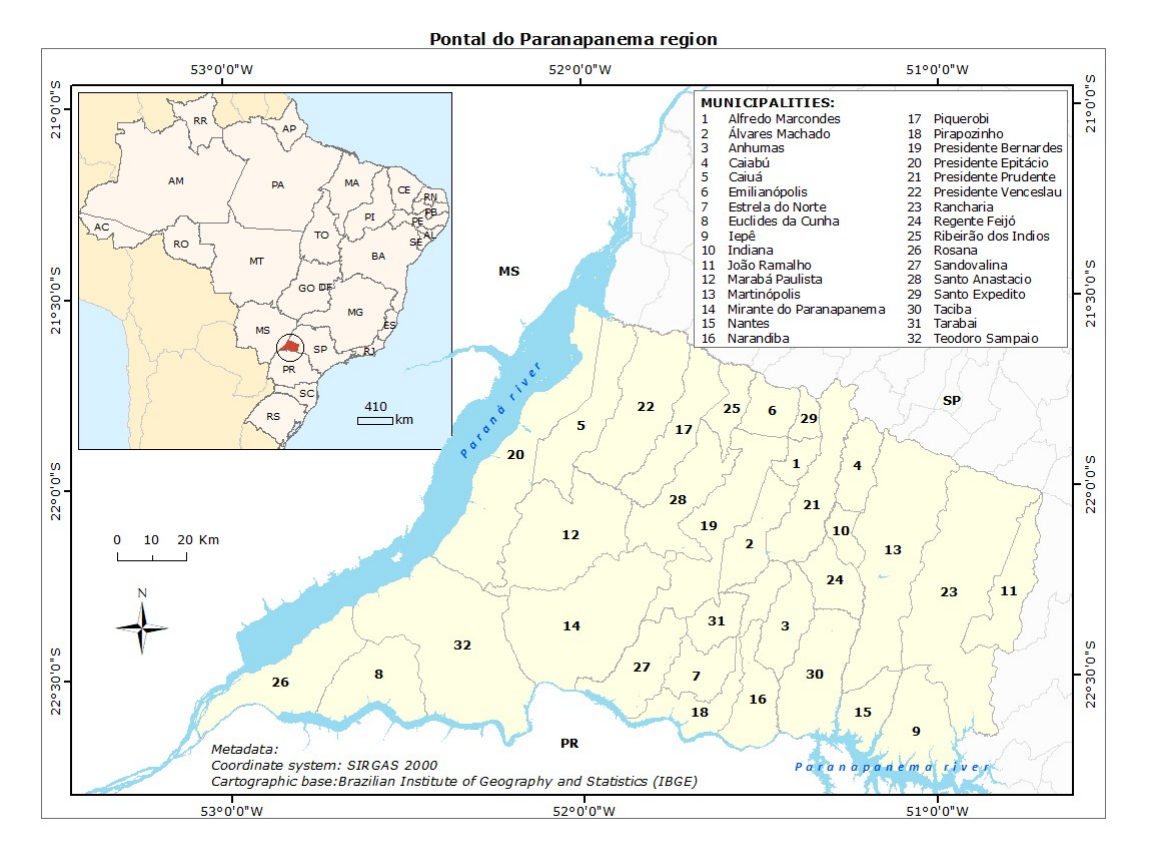
Pontal do Paranapanema location in São Paulo state and Brazil. The region is in the west side of the state, which in its turn, is located in the Southeast of Brazil.

Pontal do Paranapanema region is located in the extreme west of the state of São Paulo, southeastern Brazil, with a total area of 18,844 km^2^ and population of 583,703 people mostly concentrated in urban areas (89.74%) (Fig 1) [12].

Cases of GS and MTCT were collected from National System of Aggravations and Notification (SINAN), using the DATASUS database [13]. We included in this study 32 cities located in São Paulo state (Pontal do Paranapanema region), that reported or not cases of GS and MTCT of syphilis from 2007 to 2013. The incidence of gestational cases of syphilis (IGS) was calculated dividing number of cases (CN) notified each year by number of women of each city, while the incidence of MTCT (IMTCT) was calculated dividing number of cases reported each year by number of live births (LB) born each year in each municipality. Both incidences were standardized to 10,000 (standardized incidence rate). Data of number of women and live births were collected from Brazilian Institute of Geography and Statistics (IBGE) [12].

Incidence rate of gestational syphilis (IGS)

$$IGS=\frac{CN}{women}x\;10,000$$

Incidence rate of mother-to-child transmission (IMTCT) of syphilis

$$IMTCT=\frac{CN}{LB}x\;10,000$$

The state incidence rate was calculated by adding the average incidence of each city in the state of São Paulo, excluding 32 located in Pontal do Paranapanema and, dividing by the number of cities (614 cities) included in the calculation). This result was used to compare rates of GS and MTCT in the cities of the Pontal do Paranapanema region and the remaining regions in the state of São Paulo.

Data related to social characteristics of each city were collected from IBGE [12]. Among the variables were territorial extension, number of inhabitants, presence of fluvial areas, poverty index and the nature of health service offered to the population (public or private). In addition, numbers of settlements and lots in each city were obtained from the website of the Institute of Lands of São Paulo state (ITESP) [14]. To identify male prisons units in the Pontal do Paranapanema, the Department of Penitentiary Administration (SAP) database was consulted [15].

Characteristics about pregnant women and newborns diagnosed with syphilis were collected from DATASUS [13]. For pregnant women diagnosed with gestational syphilis variables about ethnicity (white or black), clinical classification of disease (latent, primary, secondary or tertiary), tests (treponemal or non-treponemal), educational level and residence location (rural or urban area) were collected. Regarding to newborns diagnosed with MTCT we identified variable about mother’s prenatal tests (if performed or not), pregnancy period of diagnosis (during prenatal, labor or post childbirth), treatment of partner (if performed or not) and residence location (rural or urban area).

In order to verify whether the incidence rate of MTCT presented a correlation with GS rate, in the period from 2007 to 2013, we used Pearson Correlation coefficients, tested for the hypothesis, statistically different from zero. All analyzes were performed using R [16] software and adopting a 5% level of significance.

### Seasonal and control charts

Control charts were developed for identification of seasonal trends [17], constructed in steps, as previously described [18]. First, arithmetic mean of incidence of each month individually in each year (2008 to 2013 for GS and 2007 to 2013 for MTCT was calculated (Seasonal Index–SI). Calculus of SI was made by adding Monthly Average Incidence (MAI) of cases divided by years in which there were monthly notifications published in SINAN, according to the following equations.

Gestational Syphilis:

$$SI=\frac{MAIjan\;2008+MAIjan\;2009\ldots MAIjan\;2013}{6\;years}$$

MTCT of Syphilis:

$$SI=\frac{MAIjan\;2007+MAIjan\;2008\ldots MAIjan\;2013}{7\;years}$$

The year of 2007 was not included for calculate seasonal index of GS and, consequently, construction of control chart, due to the absence of monthly information about this year on the database.

The next step to construct the control chart was to identify alert and control limits of reported cases. Firstly, the annually standard deviation (σ) of SI was calculated. Secondly, monthly Upper Alert Limit (UAL), Lower Alert Limit (LAL), Upper Control Limit (UCL) and Lower Control Limit (LCL), were calculated using the following equations:

UpperAlertLimit:UAL=SI+(2*σ)

LowerAlertLimit:LAL=SI−(2*σ).

UpperControlLimit:UCL=SI+(3*σ)

LowerControlLimit:LCL=SI−(3*σ)

When results for LAL and LCL presented negative values, those were considered equals to zero [18].

### Spatial distribution of cases

Thematic maps representing the spatial distribution of incidence rates were designed according to previous reports [19], using a Geographic Information System software (GIS), based on cartographic vector available by IBGE. The bases used consisted on 1) political-administrative limits of the cities belonging to the Pontal do Paranapanema; 2) cartography based on hydrography in the region, both in shapefile format.

## Results

### Data and statistics analyses

According to DATASUS, a total of 14,849 cases of GS and 8,365 cases of MTCT were notified in São Paulo state. Eighty cases of GS were notified in 14 out of 32 cities located on the Pontal do Paranapanema region and 61 cases of MTCT of syphilis were notified in 15 cities. Statically, incidence rate of GS was lower in Pontal do Paranapanema than the other regions in São Paulo state in 2007, 2008, 2012 and 2013 (p<0.050) and equal in 2010 and 2011. MTCT incidence rate in Pontal was higher in 2009 but similar to the other regions in the other years investigated.

**Fig 2:**
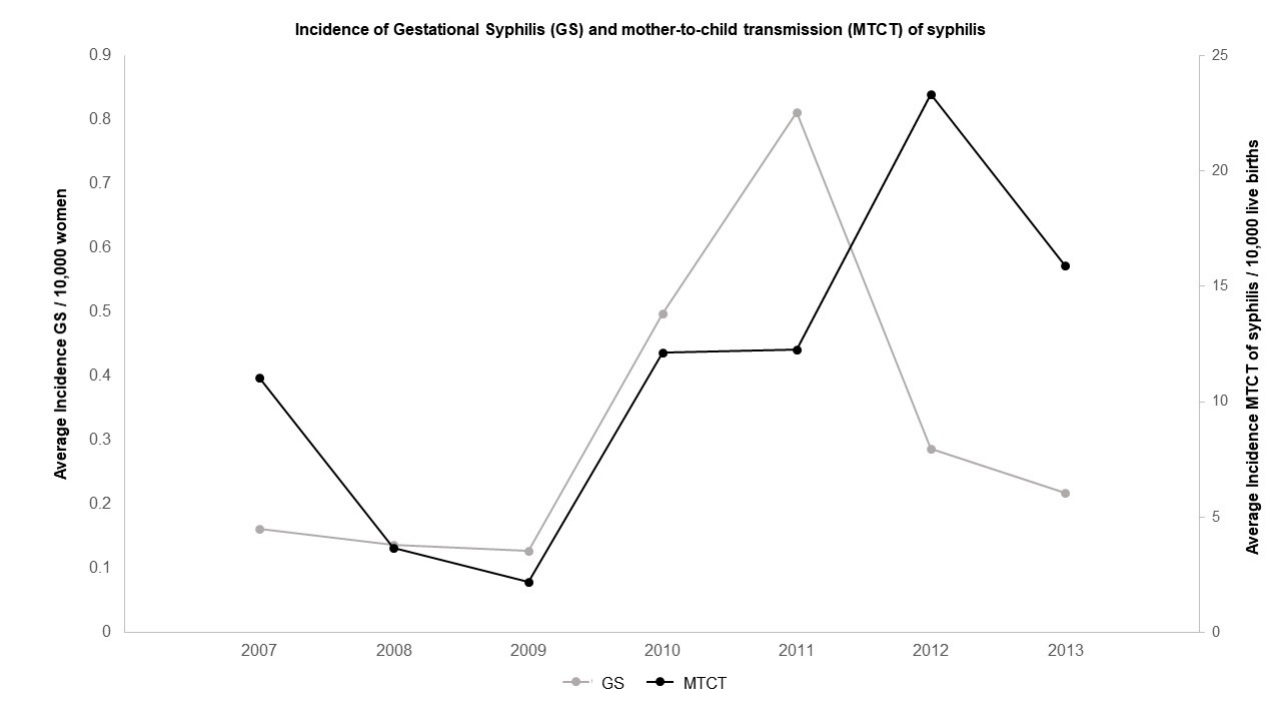
Dispersion graph showing the standardized average incidence of gestational syphilis (GS) per 10,000 habitants and mother-to-child transmission (MTCT) of syphilis per 10,000 living births reported by Pontal do Paranapanema cities from 2007 to 2013. Notification of cases increased after 2009 with peak of GS in 2011 and MTCT of syphilis in the subsequent year (2012). Database: 1) population: Brazilian Institute of Geography and Statistics—IBGE (2010); 2) notified cases: National System of Aggravations and Notification–SINAN (2017).

Incidence rate of GS increased exponentially after 2009 until 2012 in Pontal do Paranapanema, with the highest rate determined in 2011. The incidence rate of MTCT of syphilis was relatively higher when compared with GS during the period studied, reaching the peak in 2012. For MTCT of syphilis this value ranged from 3.67 to 23.29 while GS from 0.12 to 0.81. A tendential increase of rates as of 2009 was presented in both cases, with subsequent peak in 2011 to GS and 2012 to MTCT (**Fig 2)**. Correlation between GS and MTCT incidence rates in the region was significative among the months analyzed (p = 0.002) **(S1 Fig, S2 Table)**.

### Seasonal and control charts

**Fig 3:**
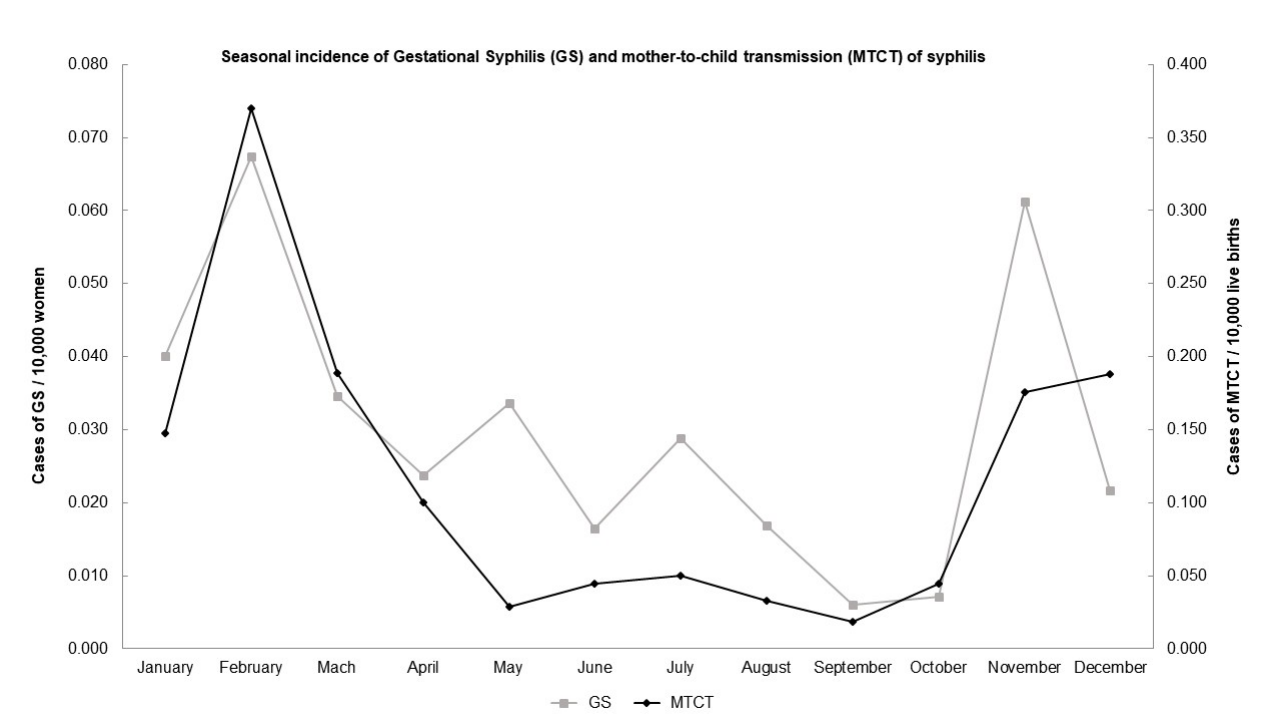
Dispersion graph showing the seasonal incidence of gestational syphilis (GS) from 2008 to 2013 in the Pontal do Paranapanema cities and mother-to-child transmission (MTCT) of syphilis from 2007 to 2013. Year of 2008 was excluded for GS because of the lack of monthly information. Highest number of cases were reported in February for both GS and MTCT of syphilis, followed by November for GS (late Spring and Summer). On the contrary, from March to October (Autumn and Winter) cases of GS and MTCT of syphilis did not reach half of February’s notifications. Database: 1) population: Brazilian Institute of Geography and Statistics—IBGE (2010); 2) notified cases: National System of Aggravations and Notification–SINAN (2017).

Seasonal pattern showed higher number of cases reported during late Spring and Summer (between November and February). Lower incidence of cases was noticed during the Autumn and Winter (between March and October) (**Fig 3)**.

**Fig 4:**
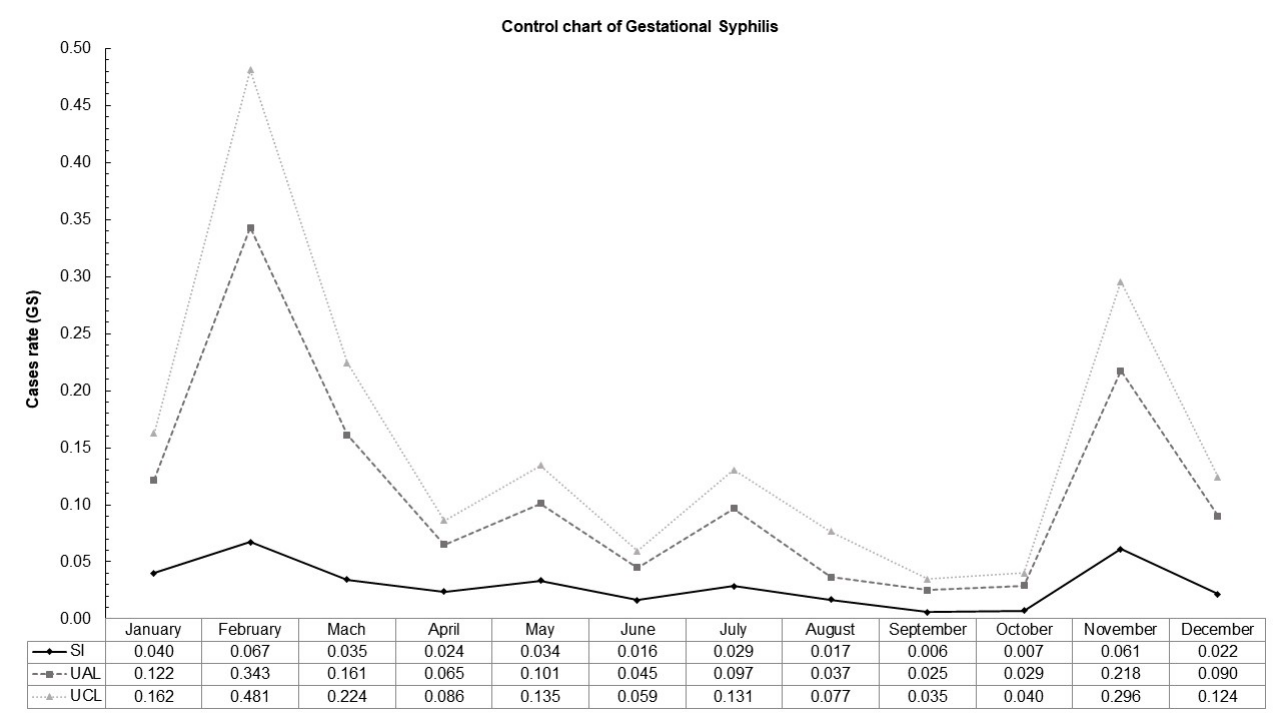
Control chart of monthly cases of gestational syphilis notified in the Pontal do Paranapanema from 2008 to 2013. SI = Seasonal index; UAL = Upper Alert Limit; UCL = Upper Control Limit. Highest amplitude of cases was identified in February followed by November, without overlap of UAL and UCL. Lowest amplitude was noticed in September and October. Inferior alert and control limits are not present as that results were lower than zero. Databases: Brazilian Institute of Geography and Statistics—IBGE (2010) and National System of Aggravations and Notification–SINAN (2017).

**Fig 5:**
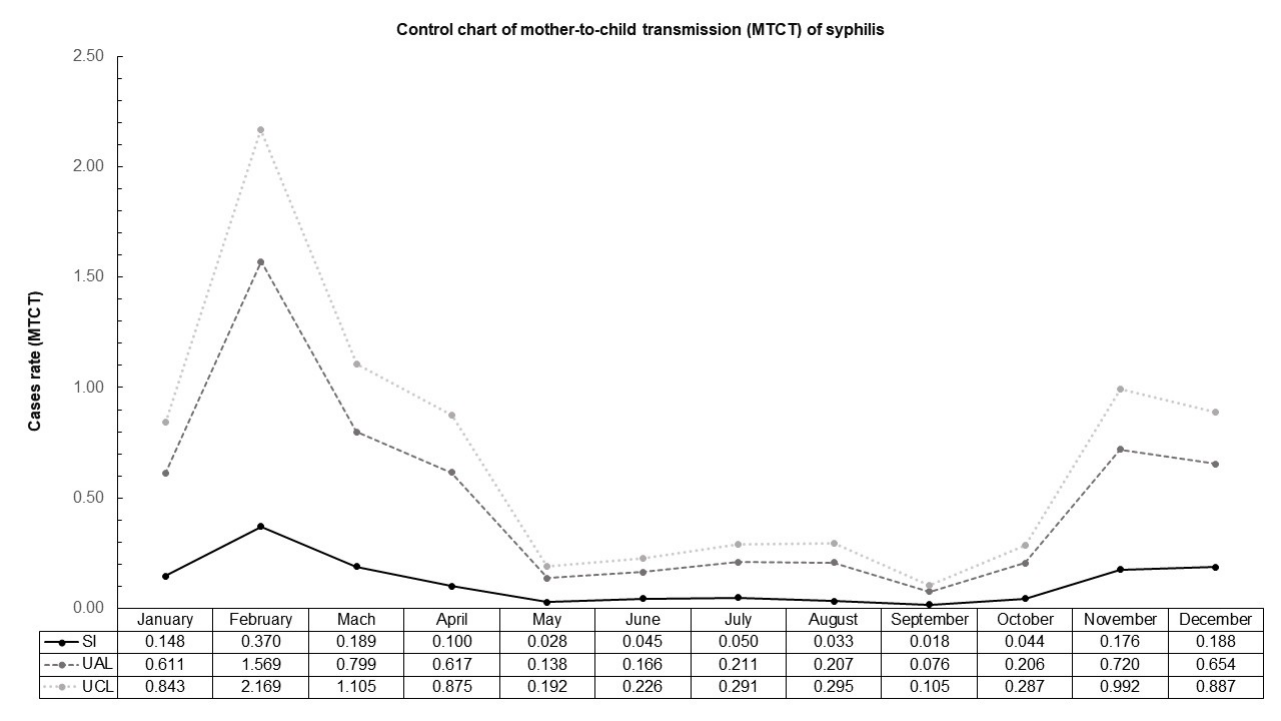
Control chart of monthly cases of MTCT of syphilis notified in the Pontal do Paranapanema from 2008 to 2013. SI = Seasonal index; UAL = Upper Alert Limit; UCL = Upper Control Limit. Highest amplitude of cases was identified in February followed by November, without overlap of UAL and UCL. Lowest amplitude was noticed in September. Inferior alert and control limits are not present as that results were lower than zero. Databases: Brazilian Institute of Geography and Statistics—IBGE (2010) and National System of Aggravations and Notification–SINAN (2017).

Our data do not contain epidemic episodes in this population, since no Upper Alert Limit (UAL) and UCL (Upper Control Limit) overlap was observed. February month presented higher amplitude indicating that during this month higher number of cases of GS and MTCT are expected to be notified. Similar pattern was observed in November with less intensity for both cases. Smaller amplitude was seen during September and October for GS whilst May and September presented smaller amplitude for MTCT **(Figs 4 and 5)**.

### Spatial distribution and population characteristics

**Fig 6:**
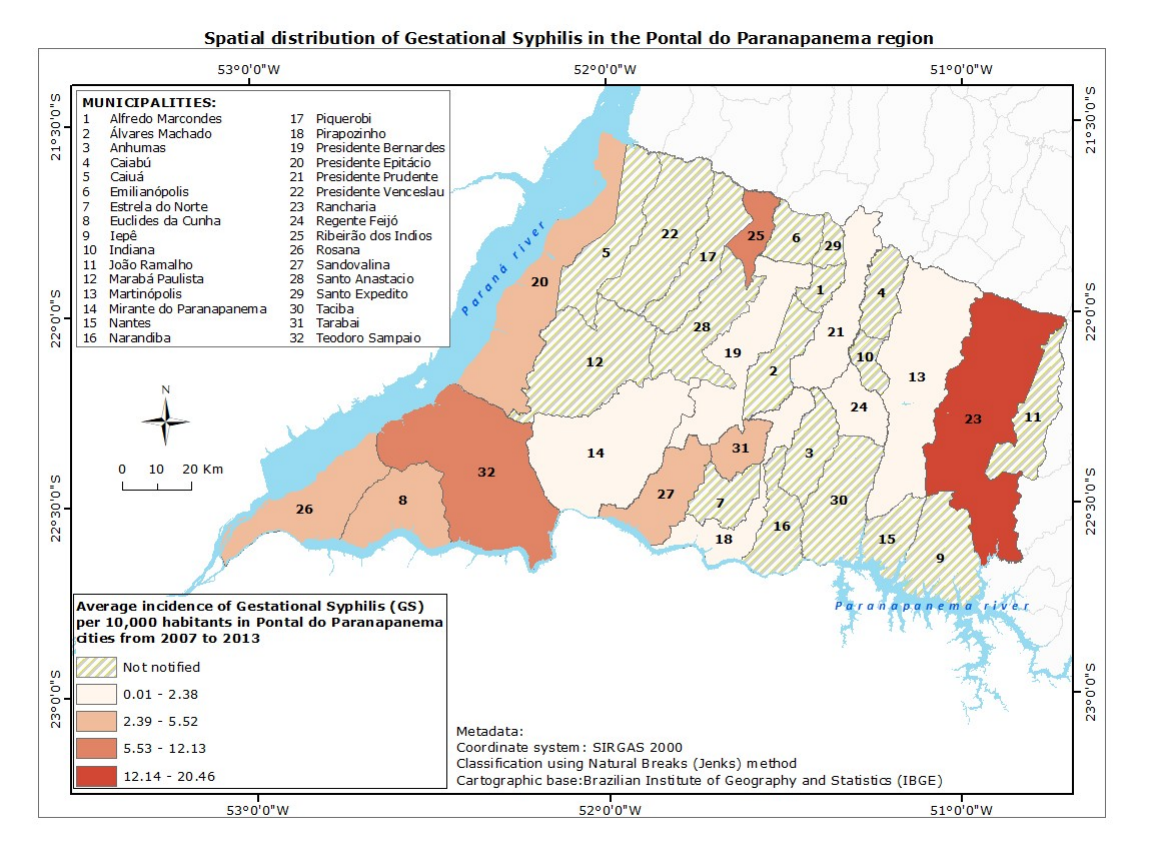
Pontal do Paranapanema cities and the spatial distribution of gestational syphilis reported cases from 2007 to 2013. The highest incidence of cases among the cities is represented by Rancharia (red), followed by Ribeirão dos Índios and Teodoro Sampaio (orange). The lowest index average incidences were identified in Martinópolis, Presidente Prudente, Presidente Bernardes e Mirante do Paranapanema (beige). Cities that reported cases of GS are located in the state border with Mato Grosso do Sul (West) and Parana (South). Databases: Brazilian Institute of Geography and Statistics—IBGE (2010) and National System of Aggravations and Notification–SINAN (2017).

**Fig 7:**
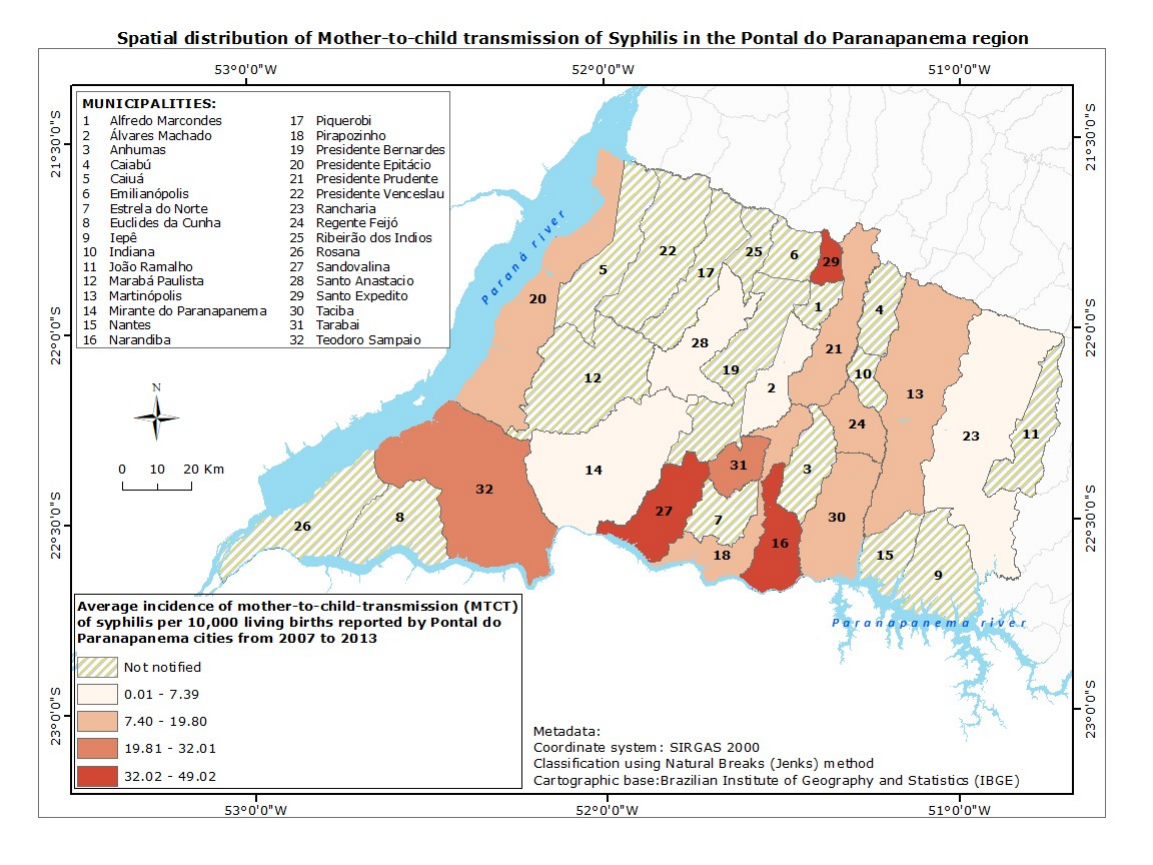
Pontal do Paranapanema cities and the spatial distribution of mother-to-child transmission (MTCT) of syphilis reported cases from 2007 to 2013. The highest incidences of cases were represented by Sandovalina, Narandiba e Santo Expedito (black) followed by Tarabai and Teodoro Sampaio (burgundy). Presidente Prudente, Regente Feijó and Martinópolis reported an index of 7.54 to 15.25 (salmon) and, the lowest index average incidences were identified in Santo Anastácio, Álvares Machado, Rancharia e Mirante do Paranapanema (salmon). Cities that reported cases of MTCT of syphilis are located mainly in the border with Parana state (South). Databases: Brazilian Institute of Geography and Statistics—IBGE (2010) and National System of Aggravations and Notification–SINAN (2017).

Aiming to analyze possible endemic outbreaks, we found that higher concentration of reported cases of GS and MTCT was observed in cities in the border between Pontal do Paranapanema and Mato Grosso do Sul state, as well as in the Pontal and Parana state. Incoherence of notification was noticed in Narandiba, Taciba, Santo Anastácio, Santo Expedito and Álvares Machado cities, since there was notification of MTCT in the absence of notification of cases of GS. The cities of Euclides da Cunha Paulista, Rosana and Ribeirão dos Índios presented absence of MTCT cases even in the presence of cases of OS, suggesting a better control of transmission to the fetus/neonate. Distribution of reported cases of GS and MTCT, as well as incidence rate of cases, are shown in Figs **6 and 7**, respectively.

Variables of interest including infrastructural socioeconomic and health characteristics of the city were analyzed to better characterize incidence of GS and MTCT (Table 1). Primarily, our hypothesis was that women residing in rural settlements had more chance to be part of the underreported cases, since those places are located generally in the peripheral area of city with no health services nearby. However, we have noticed that cities with rural settlements reported cases of GS and no cases of MTCT, suggesting better approach to prevent MTCT of syphilis. In addition, there were no correlations between the poverty index, nature of the health service and incidence of reported case.

**Table 1:** Variables related to cities where cases of gestational syphilis (GS) and mother-to-child transmission (MTCT) of syphilis were notified. Álvares Machado, Narandiba, Pirapozinho, Presidente Prudente, Regente Feijo, Santo Anastácio Santo Expedito and Taciba did not present rural settlements. No statistics correlation was found between poverty index or the nature of the health service with the incidence of reported cases. Databases: *Brazilian Institute of Geography and Statistics—IBGE (2010); ** Institute of Lands of São Paulo state–ITESP (2017).

City	Territorial Extension (km^2^)*	Population*	River areas*	Allotment / Settlement**	Poverty rate (%)*	Public health services*	Private health services*
Álvares Machado	347,646	24,651	No	0	27.25	5	2
Euclides da Cunha Paulista	573,894	9,642	Yes	9/1041	46.86	5	1
Martinópolis	1,253,564	25,805	Yes	2/124	31.96	9	2
Mirante do Paranapanema	1,238,931	17,979	Yes	31/1326	34.72	10	1
Narandiba	357,325	4,657	No	0	32.17	4	0
Pirapozinho	477,763	26,594	Yes	0	29.24	7	5
Presidente Bernardes	749,234	13,568	Yes	8/266	17.59	7	2
Presidente Epitácio	1,260,281	43,535	Yes	4/388	31.31	10	13
Presidente Prudente	560,637	207,610	Yes	0	14.47	39	68
Rancharia	1,587,498	29,778	Yes	2/199	28.59	10	6
Regente Feijó	263,28	19,733	No	0	22.52	8	3
Ribeirão dos Índios	196,446	2,245	No	1/40	27.22	1	0
Rosana	744,011	18,459	Yes	3/201	13.35	12	5
Sandovalina	455,856	4,076	Yes	2/198	28.87	2	0
Santo Anastácio	552,876	20,475	Yes	0	27.18	5	4
Santo Expedito	94,465	2,803	No	0	39.50	1	0
Taciba	607,267	5,714	Yes	0	39.21	3	0
Teodoro Sampaio	1,555,803	21,386	Yes	20/849	37.33	13	5

Although there are not female prison units in the Pontal do Paranapanema, we decided to investigate whether high population rate in male prison units would be related to GS or MTCT cases in the cities investigated, since transmission of syphilis to women can occur in those institutions during intimate visits. Our results showed that the highest rate of prisoners per habitants were in Marabá Paulista e Caiuá cities (>20%) (Table 2). Both cities did not reported GS or MTCT cases in the period investigated. Presidente Venceslau, Martinópolis and Presidente Prudente presented cases of GS and MTCT with similar demography of prisoners per habitants, 8.2 and 9.4% respectively. Thus, we concluded that male overpopulation in prison units is not related to transmission of gestational or MTCT of syphilis in Pontal do Paranapanema region.

**Table 2:** Population in male prison units located in the Pontal do Paranapanema region. All prisons showed number of prisoners higher than its maximum capacity, excepted Presidente Bernardes city. Range of prisoners per habitants variated from 4.10 to 33.60. Databases: *Brazilian Institute of Geography and Statistics—IBGE (2010); ** Department of Penitentiary Administration—SAP (2018).

City	Population*	Maximum capacity**	Total of prisoners**	Prisoners per habitants (%)**
**Caiua**	5,039	844	1,120	22.20
**Maraba Paulista**	4,182	844	1,409	33.60
**Martinópolis**	24,219	872	1,990	8.20
**Presidente Bernardes**	13,570	1,451	2,175	16.00
**Presidente Prudente**	207,610	943	1,877	9.40
**Presidente Venceslau**	37,910	2,061	1,557	4.10

Important variables were identified during analysis of children diagnosed with MTCT syphilis. Surprisingly, 95% of child diagnosed with MTCT of syphilis were born from mothers enrolled in pre-natal care that included screening for syphilis. Our data also has shown that 54.10% of mothers received the diagnose of GS during pre-natal care while 45.90% were diagnosed in intrapartum and postpartum periods. A total of 93% of mothers declared to live in urban areas and 65% of partners having sexual relationship with pregnant women has not conducted the correct treatment indicate by physician after diagnosis of syphilis (Table 3). Other variable found in MTCT of syphilis diagnosis, related to gender, mother’s pre-natal care, diagnosis of gestational syphilis and prognostic are shown on Table 3.

**Table 3:** Variables related to cases of mother-to-child transmission (MTCT) of syphilis notified from 2007 to 2013 in cities located in the Pontal do Paranapanema region. A total of 61 cases of MTCT of syphilis were notified. In 58 of those (95.08%) mother was assistance during pre-natal period and 33 (54.10%) were diagnosed with gestational syphilis (GS) at this period. Diagnosis in intrapartum and postpartum of GS were, respectively, determined in 42.62% and 3.28% of the women in the region. None of cases of MTCT of syphilis lead to death in this region. Database: National System of Aggravations and Notification—SINAN (2017).

Variable		Notified cases (n = 61)	Percentage (%)
**Gender**	*Female*	31	50.82
	*Male*	30	49.18
**Mother’s prenatal care**	*Yes*	58	95.08
	*No*	3	04.92
**Diagnose of gestational syphilis**	*Prenatal*	33	54.10
	*Intrapartum*	26	42.62
	*Postpartum*	2	03.28
**Concurrent partner treatment**	*Yes*	16	26.23
	*No*	40	65.57
	*Missing*	5	08.20
**Residence location**	*Urban*	57	93.44
	*Rural*	4	06.56
**Prognosis**	*Deaths*	0	00.00

We analyzed data from pregnant women diagnosed with syphilis who gave birth to a child with syphilis as well as the type of diagnosis test performed. From a total of 80 cases in the region, the most common syphilis clinical stages were secondary, primary and latent syphilis, in order of appearance. Non-treponemal test (screening test with higher sensibility) was positive in 96% of the cases while treponemal test (confirmatory test with higher specificity) was positivein 66% of them. Eighteen percent of the mothers were not submitted to a confirmatory treponemal test. Most mothers diagnosed with GS (86.25%) presented low educational level and had attended school for less than 10 years **(Table 4)**.

**Table 4:** Variables related to mothers diagnosed with gestational syphilis (GS), from 2007 to 2013 living in cities located in the Pontal do Paranapanema region. A total of 80 cases of GS were notified by cities analyzed. Pregnant women affected by syphilis were mostly white (68.75%). Fifty percent of cases were diagnosed during secondary stage, followed by primary (17.50%), next latent (13.75%), then tertiary (5%). Non-treponemal test (screening test) was reactive to 96.25% of pregnant women infected whilst Treponemal test (confirmatory test) was reactive to 76.25% and not performed in 18.75% of late confirmed cases. Educational level was related to GS occurrence in this population, since 86.25% of them studied less than 10 years. Most of pregnant women lived in urban areas (92.45%). Database: National System of Aggravations and Notification–SINAN (2017).

Variable		Notified Cases n = 80	Percentage (%)
**Ethnicity**	*White*	55	68.75
	*Black*	2	5.00
	*Brown/Mixed race*	21	26.25
**Clinical classification of syphilis**	*Primary*	14	17.50
	*Secundary*	40	50.00
	*Tertiary*	4	5.00
	*Latent*	11	13.75
	*Missing*	11	13.75
**Non-treponemal test**	*Reactive*	77	96.25
	*Non-reactive*	1	1.25
	*Not tested*	2	2.50
**Treponemal test**	*Reactive*	61	76.25
	*Non-reactive*	4	5.00
	*Not tested*	15	18.75
**Educational level**	*< 8 years*	41	51.25
	*Collegue degree*	28	35.00
	*Superior degree*	1	1.25
	*Missing*	10	12.50
**Residence location**	*Urban*	74	92.50
	*Rural*	6	7.50

## Discussion

In this study, we identified the epidemiological profile of syphilis in both maternal and newborn population in the Pontal of Paranapanema region. Despite accessible diagnosis and treatment, cases of syphilis are still increasing in Brazil, suggesting a failure to control and eliminate this STI. Recent data, made available by Epidemiological Surveillance agencies, indicates a worrying increase in the number of cases of GS and MTCT throughout the country [5,20,21]. Therefore, innovative approaches are needed to understand the dynamics of this disease in the population. Study of temporal variations on infectious diseases allows the understanding of the transmission dynamics in a certain population [22]. For this evaluation, we used the seasonal index, which consists of graphic sampling from the behavior of the disease over a period of time, and the control diagram that allows the identification of variations and mathematical limits for the prediction of endemic episodes [23].

Brazil's epidemiological surveillance uses a large scale control chart to understand the dynamics of infectious diseases [17,24], mainly to comprehend dynamics of vector diseases that undergoes direct influence from climate change and then are classified as seasonal diseases [25]. Regarding gestational and mother-to-child transmission of syphilis, no studies were found in the literature using this tool to understand the disease dynamics, thus leading to the innovative approach in our study.

To identify epidemic episodes of GS and MTCT, we analyzed cases reported monthly from 2007 to 2013 in cities located in the Pontal do Paranapanema. Analysis of control chart allows the identification of endemic peaks when the index is greater than the Upper Control Limit. The alert thresholds may indicate a biased increase to a period of epidemic, leading to the health surveillance system to take precautions in order to avoid an epidemy [17,26]. In our study, we did not observe outbreaks since neither GS nor MTCT showed overlap of limits.

Araujo and colleagues,based on observational and variational temporal analysis of cases reported in Brazil, found a MTCT rate ranging from 1.7 to 2.10 per 1,000 live births in the years 2003 to 2008 [27], very close to the values identified in our study, from 0.37 to 2.30 (2007 to 2013). They also reported a GS detection coefficient of 2.5 for every 1,000 inhabitants during 2003 to 2008, higher than our result from 0.12 to 0.81 for 10,000 inhabitants.

The significant increase in cases of gestational syphilis observed from 2011 may be related to the implementation of the national program "*Rede Cegonha*" and a consequent increase in cases detected. This program established periodic tests for syphilis detection during pre-natal, intrapartum and postpartum. In the latter, the newborn is assisted until two years old. In addition, a strategic agenda of the Epidemiological Surveillance was elaborated for the years 2011 to 2015, with the objective of reaching 100% of the pregnant women undergoing prenatal care undergoing the syphilis test [28,29].

We suggest a failure in the GS cases notification system, since cities that reported MTCT cases did not necessarily report GS cases. This bias can explain the low rates of GS cases and the overall difficult to effectively screen the disease in the country. This observation has been previously reported by Komka e Lago (2007), which identified 64% of notification rate for MTCT of syphilis in a reference hospital in the state of Tocantins, Brazil [30].

Failure to correctly diagnose syphilis during pregnancy culminates in non-treatment or inadequate adherence to treatment [31]. The inadequate treatment of GS cases reported in 2011 may have influenced the significant increase in SC cases in the year 2012, which corroborates the data reported by Muricy e Pinto (2015). According to them, 87.2% of the pregnant women living in the Federal District receive adequate prenatal care and 52.6% were diagnosed with syphilis. Only 22% of infected pregnant women were adequately treated and 24.8% of the partners, which makes reinfection possible even after treatment, increasing the chances of transmission of syphilis to the fetus [32].

Using analysis of alert range and case control limits, we determined that the month with the highest number of cases was February. Previously in Brazil, Passos and collegues (2010) investigated whether Carnival could influence the transmission and the number of cases of STIs. According to the authors, there was no correlation between the transmission and number of cases of STIs during the carnival period[33]. February is also summer time in the country and infectious diseases rates increase in warm and rainy periods in Brazil as the vector multiply better in this period. Then, more people seek for medical assistance, increasing diagnosis of infectious diseases [34]. Another important factor is the increase of travel during summer vacation, when people could potentially increase transmission of *T*. *pallidum* by sexual intercourse [35]. Finally, public campaigns against syphilis in the country are held on November and February. Those campaigns promote strategies of diagnosis and detections, then it also may explain the higher rate of detection in February. However, we have not observed any correlations between incidence of syphilis and the entire summer in the period analyzed, even considering that *T*. *pallidum* develop symptoms, at least, 30 days after infection [36].

There were discrepancies between reported cases of GS and MTCT, suggesting incorrect treatment or misdiagnosis of syphilis during gestational period. In the cities of Narandiba (2011 e 2012), Álvares Machado (2010), Santo Anastácio (2012), Santo Expedito (2013) and Taciba (2010) cases of MTCT were reported, but no cases of GS were registered.

Between 2010 and 2013 underreported cases of GS in Pontal do Paranapanema reached 13.74%. Underreporting of gestational syphilis cases in Brazil is a common fact already reported by other authors [37,38]. Komka and Lago (2007), emphasizes that MTCT of syphilis is also affected by underreporting because of late diagnosis of GS leads to high mortality rates for MTCT [30].

Our data suggest a failure in the diagnosis and/or treatment of GS in the population from 2007 to 2013. In more than 90% of MTCT cases the mothers had access to prenatal care. Only half of the mothers were diagnosed with GS during the prenatal care period (54.10%). Another sizable portion was diagnosed later, during delivery or curettage (42.62%). Prenatal care coverage is an important factor in the diagnosis and consequently the late search for prenatal care during pregnancy may lead to an increased risk of syphilis transmission to the fetus [39,40]. Therefore, although almost 100% of the pregnant women who had children diagnosed with MTCT of syphilis in the Pontal do Paranapanema have been attended by prenatal care, not all of them were diagnosed early. Hence, the difficulty of treatment and infection of the fetus is not limited to screening errors, but also limitation on search for health care service by pregnant women.

Among the children diagnosed with MTCT, more than 65% were not submitted to any treatment. This important finding agrees with previous studies highlighting the importance of treating the partner to avoid reinfection of the mother and consequently to the fetus [40,41]. In a study performed in 2014 in Porto Alegre city, located in South of Brazil, it was found that less than 50% of the partners are treated for syphilis and there were limitations on the notification of treatment [42]. As a consequence of this behavior, reinfection of mothers can occur at any time during pregnancy, being most worrying during last weeks of pregnancy, when there is no more time for treatment before delivery, despite the stage maternal disease [43].

Most of the mothers diagnosed with GS in the present study are white. This finding reinforces the need for studies focused on the distribution and analysis of population considering that different regions may present singular profiles and that previous reports showed high prevalence of gestational syphilis among non-white women [44]. In addition, is important to consider that more than 60% of the Brazilian population is comprised by mixed/brown and black people. Subsequently, analysis of these profiles may assist in better tracking and planning effective approaches to control and elimination of syphilis.

Reported cases of gestational and MTCT of syphilis identified in this study express a fragility in the notification system, since cities notifying MTCT do not necessarily report syphilis in pregnant women. Therefore, it is necessary to improve the reporting system so the data can provide better conditions to monitor and control transmission of syphilis.

The use of the tools presented here can contribute to improve health strategies aimed to prevent and control syphilis, as well as other sexually transmitted infections in pregnant women in Brazil.

## Supporting information

S1 Table Notified cases of gestational syphilis and incidence rate per 10,000 women in all São Paulo municipalities. Pontal do Paranapanema cities are highlighted in gray.
(XLSX)

S2 Table Notified cases of mother-to-child transmission (MTCT) of syphilis and incidence rate per 10,000 live birth in all São Paulo municipalities. Pontal do Paranapanema cities are highlighted in gray.
(XLSX)

S3 Table Comparison between incidence of GS and MTCT of syphilis in Pontal do Paranapanema from 2007 to 2013. a) Comparison between municipalities; b) GS and MTCT in the region, per month c) GS and MTCT in the region, per year
(XLSX)

S1 Fig Correlation between incidence of GS and MTCT of syphilis in Pontal do Paranapanema from 2007 to 2013. (TIF)
