# Supplementary figures and images for: Mother-to-child transmission and gestational syphilis: Spatial-temporal epidemiology and demographics in a Brazilian region
Source: PLoS Negl Trop Dis. 2019 Feb 21;13(2):e0007122. doi: 10.1371/journal.pntd.0007122 (PMC6383870; doi:10.1371/journal.pntd.0007122)

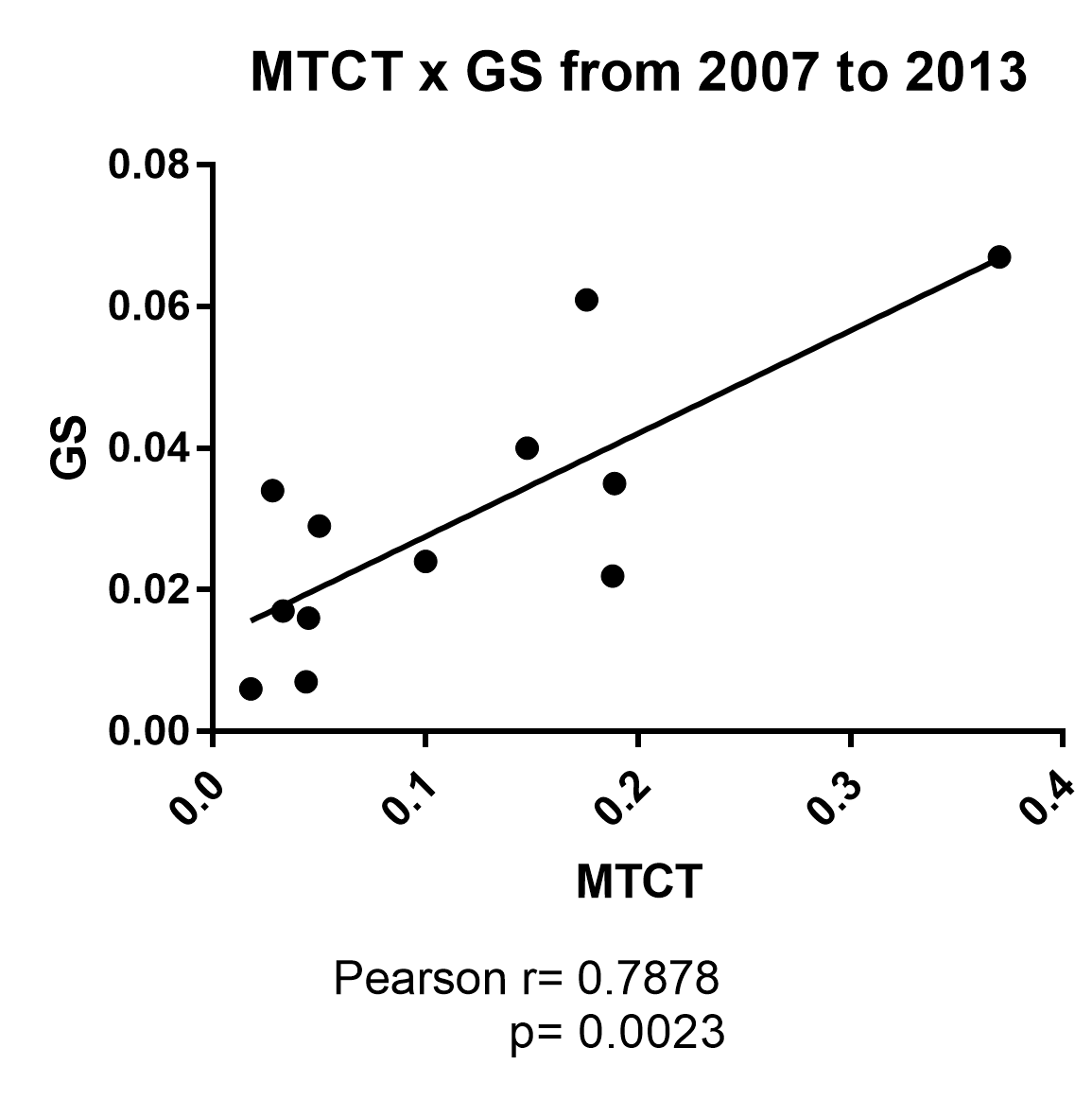

Supplement: S1 Fig — (TIF) [file pntd.0007122.s004.tif]
